# Trends in Total Joint Arthroplasty Among Patients With Rheumatoid Arthritis: The Effect of Recent Disease Modifying Antirheumatic Drug Utilization Guidelines

**DOI:** 10.5435/JAAOSGlobal-D-22-00209

**Published:** 2022-12-05

**Authors:** Thomas W. Hodo, J. Heath Wilder, Bailey J. Ross, Matthew W. Cole, Felix H. Savoie, William F. Sherman

**Affiliations:** From the Department of Orthopaedic Surgery, Tulane University School of Medicine, New Orleans, LA (Dr. Hodo, Dr. Wilder, Dr. Cole, Dr. Savoie, and Dr. Sherman), and the Department of Orthopaedic Surgery, Emory University School of Medicine, Druid Hills, GA (Dr. Ross).

## Abstract

The 2015 change in the American College of Rheumatology (ACR) guidelines narrowed indications for initiating treatment with biologic disease-modifying antirheumatic drugs (bDMARDs) in patients with rheumatoid arthritis (RA). This study sought to evaluate trends in total joint arthroplasty (TJA) in patients with RA and to characterize the effect of bDMARDs on arthroplasty risk in this population after the change in ACR treatment guidelines.

**Methods:**

A retrospective review was conducted using the PearlDiver database. TJA procedures included total shoulder arthroplasty, total elbow arthroplasty, total hip arthroplasty, and total knee arthroplasty. The Cochran–Armitage Trend Test was used to evaluate trends in the volume of TJA procedures conducted in patients with RA between 2010 and 2019. Logistic regression was used to compare 2-year arthroplasty risk after an initial joint-specific RA International Classification of Diseases 10th Revision diagnosis for RA patients with versus without bDMARD exposure.

**Results:**

A total of 2,942,360 patients with RA were identified, and 80,744 (2.74%) underwent TJA between 2010 and 2019. Rates of TJA procedures trended significantly upward over the decade (2.6% versus 5.1%, *P* < 0.001) with a sharp increase between 2015 and 2016 (2.1% versus 4.9%, *P* < 0.001). Among the 16,736 identified patients with an initial International Classification of Diseases 10th Revision joint-specific RA diagnosis, 3362 patients (20.09%) were treated with bDMARDs and 13,374 (79.91%) were not. Untreated patients exhibited significantly lower risk of any TJA (5.92% versus 7.73%; odds ratio [OR]: 0.72; 95% confidence interval [CI]: 0.64 to 0.82), total hip arthroplasty (OR: 0.69, 95% CI: 0.50 to 0.95), and total knee arthroplasty (OR: 0.63, 95% CI: 0.52 to 0.75) compared with treated patients.

**Discussion:**

The volume of TJA procedures conducted in patients with RA has trended markedly upward over the past decade, with a sharp increase after 2015. bDMARD treatment was associated with markedly increased risk of TJA, likely because of initiation of bDMARDs in only those patients with advanced disease per ACR guidelines.

Rheumatoid arthritis (RA) is an autoimmune, inflammatory variant of degenerative joint disease characterized by lymphocyte and monocyte invasion of the synovium with eventual involvement of the periarticular bone that results in cartilage and bony erosion.^1^ RA has a prevalence of approximately 0.5% in the United States and is two to three times more common in women, with peak prevalence in patients aged 40 to 60 years.^2,3^ Most patients initially experience swelling and pain of the smaller joints, such as the hands, with additional progression that typically involves the larger joints, such the knees, hips, and shoulders.^4^ Progressive joint degeneration, laxity due to tendon/ligament involvement, and deformity ensue with disease progression.^4^

Advancements in RA treatment with disease-modifying antirheumatic drugs (DMARDs) have markedly improved symptoms and functionality in patients with RA.^1,5^ In 1998, biologic DMARDs (bDMARD), such as etanercept and infliximab, were introduced.^6,7^ Both these tumor necrosis factor alpha inhibitors (TFNais) have been demonstrated to slow RA disease progression or prevent joint degeneration altogether when initiated early in the RA disease process.^6,7^ As a result, the need for total joint arthroplasty (TJA) in patients with RA declined considerably after widespread utilization of bDMARDs, especially among young patients.^8^ However, recent American College of Rheumatology (ACR) guidelines (2012; 2015; and 2021) do not advocate for bDMARD utilization in early disease as first-line treatment because of associated health risks (e.g., infection and malignancy) and high cost of medication. This recommendation contrasts to the prior 2008 ACR guidelines.^4,9,10,11,12^

Given the recent change in ACR guidelines, the effect on TJA utilization in patients with RA in recent years is unclear. The purpose of this study was to characterize trends in TJA utilization in patients with RA in the United States between 2010 and 2019 and analyze the effect of bDMARD exposure on two-year arthroplasty risk. In light of the potential reduction in TJA utilization in the years after the introduction of bDMARDs, we hypothesized that the recent change in ACR guidelines would be associated with upward trends in TJA utilization in patients with RA.^13^ In addition, we hypothesized that RA patients with bDMARD exposure would exhibit markedly lower risk of TJA.

## Methods

### Data Source

A commercially available nationwide administrative claims database, PearlDiver-Mariner patient claims database (PearlDiver Technologies), containing 144 million patients was used to retrospectively review deidentified patient records. This study used the ‘M91Ortho’ data set within PearlDiver, which contains a random sample of 91 million patients with diagnoses or procedures pertaining to the musculoskeletal system across multiple insurance provider groups in US territories and states including commercial insurance groups, Medicare, and Medicaid from 2010 through Q3 of 2020. The Current Procedural Technology and International Classification of Diseases Ninth Revision and Tenth Revision (ICD-9/ICD-10) codes were used to identify patients and define outcomes. Institutional review board exemption was granted through our institution because the provided data were deidentified and compliant with the Health Insurance Portability and Accountability Act. No outside funding was received for this study.

### Patient Selection and Outcomes

Patients with a diagnosis of RA between 2010 and 2019 were identified in the database using ICD-9 and ICD-10 diagnosis codes. To ensure that only patients eligible for a total joint arthroplasty were included in our study, patients younger than 40 years were excluded. Current Procedural Technology codes were used to identify patients with RA who received TJA, including total shoulder arthroplasty (TSA), total elbow arthroplasty (TEA), total hip arthroplasty (THA), and total knee arthroplasty (TKA). Patients who underwent arthroplasty procedures before being diagnosed with RA were excluded. Volume of TJAs in patients with RA was queried for each year between 2010 and 2019.

To analyze the effect of bDMARD exposure on risk of arthroplasty, patients with RA-associated diagnoses localized to the shoulder, elbow, hip, or knee joint were identified. ICD-9 diagnosis codes for RA do not specify the afflicted joint(s), and therefore, only ICD-10 diagnosis codes were used to identify patients with RA in this secondary analysis. This methodology was used to identify patients with relevant joint-related pathology so that risk of arthroplasty for the affected joint could be assessed. In addition, because ICD-10 was implemented in 2015, this approach allowed for an analysis of the risk of arthroplasty after the change in ACR guidelines. Among these patients, treated patients were defined by at least one claim for a bDMARD within 2 years before or 2 years after an initial joint-specific RA diagnosis. Patients treated with medications aside from bDMARDs were defined as patients with RA without a record of a bDMARD claim within 2 years before or 2 years after an initial joint-specific RA diagnosis. Patients were only included if they had continuous database enrollment during this period to allow for evaluation of bDMARD exposure and a minimum of 2-year follow-up. Rates of TJA at 2 years after the joint-specific RA diagnosis were queried and compared for the two cohorts.

### Study Population

From 2010 to 2019, a total of 2,942,360 patients with RA were identified, and 80,744 (2.74%) of these patients underwent TJA. For each individual arthroplasty procedure, 7737 patients received a TSA (9.58%); 639 patients received a TEA (0.79%); 21,580 patients received a THA (26.73%); and 50,788 patients received a TKA (62.90%). Among the 16,736 identified RA patients with an ICD-10 joint-specific RA-related diagnosis, 3362 patients (20.09%) had a record of bDMARD treatment and a total of 13,374 patients (79.91%) did not.

### Statistical Analyses

Statistical analyses were conducted using the R statistical software (version 4.1.0; R Project for Statistical Computing) integrated within the PearlDiver software and Microsoft Excel (Microsoft Corporation) with the XLStat statistical package add-on (Addinsoft) with an α level set to 0.05. A Cochran–Armitage Trend Test was conducted to evaluate trends in the volume of TJAs conducted in patients with RA between 2010 and 2019 to test the two-tailed null hypothesis that the volume of TJA procedures in this population remained constant over the decade. Rates of TJA procedures conducted in 2015 versus 2016 were compared with chi square analysis to characterize the immediate effect of the revised ACR guidelines.

Among identified patients with ICD-10 diagnoses for joint-specific RA pathology, the anatomic distribution of diagnoses was compared for patients treated with bDMARDs versus those not treated with bDMARDs using chi square analysis. Rates of TJA within 2 years of an initial joint-specific RA-associated diagnosis were compared for bDMARD-treated versus non-bDMARD (nbDMARD) patients using multivariable logistic regression. Odds ratios (ORs) with associated 95% confidence intervals (CIs) were calculated to compare risk of any TJA procedure and each individual TJA procedure.

## Results

### Trends in Total Joint Arthroplasty

**Figure 1:**
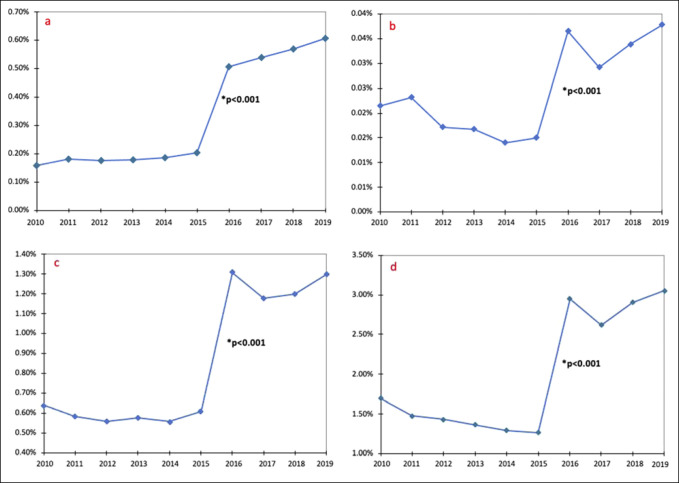
Graphs showing the trends of arthroplasty procedures in patients with RA over the past decade. **A,** TSA, (**B**) TEA, (**C**) THA, and (**D**) TKA. *P* values correspond to the change in arthroplasty volume from 2015 to 2016. RA = rheumatoid arthritis, TEA = total elbow arthroplasty, THA = total hip arthroplasty, TKA = total knee arthroplasty, TSA = total shoulder arthroplasty

Between 2010 and 2019 (Table 1)**,** the percentage of patients with RA receiving TJA procedures significantly increased from 2.55% to 5.14% (*P* < 0.001). At the individual procedure level (Figure 1)**,** utilization of TSA increased from 0.016% to 0.063% (*P* < 0.001), TEA increased from 0.022% to 0.040% (*P* < 0.001), THA increased from 0.65% to 1.35% (*P* < 0.001), and TKA increased from 1.71% to 3.11% (*P* < 0.001) (Supplemental Table 1, http://links.lww.com/JG9/A238). The period between 2015 and 2016 showed the largest uptrend in volume of arthroplasty conducted among RA patients with a 232.08% increase in total TJA volume (*P* < 0.001), 253.77% increase in TSA (*P* < 0.001), 249.51% increase in TEA (*P* < 0.001), 218.80% increase in THA (*P* < 0.001), and 234.79% increase in TKA (*P* < 0.001).

**Table 1:** Anatomic Distribution of ICD-10 Joint-Specific RA Diagnoses

Procedure	bDMARD Treatment n (%)^a^	No bDMARD Treatment n (%)^a^	*P* Value
Total	3362 (100)	13,374 (100)	—
Shoulder	1333 (39.6)	5768 (43.1)	**<0.001**
Elbow	433 (12.9)	1354 (10.1)	**<0.001**
Hip	386 (11.5)	1655 (12.4)	0.157
Knee	1210 (36.0)	4687 (35.0)	0.305

bDMARD = biologic disease modifying anti-rheumatic drug, ICD-10 = International Classification of Diseases Tenth Revision, RA = rheumatoid arthritis, TJA = total joint arthroplasty

a Percentages calculated of the total number of patients in each group (All TJA) or the number of patients from each group with pathology in the given joint.

Bolded *P* values/OR (95% CI) indicate statistically significant results.

### Total Joint Arthroplasty Rates in Patients With Biologic Disease-Modifying Antirheumatic Drugs Versus No Biologic Disease Modifying Antirheumatic Drug

Among the identified patients with ICD-10 diagnoses for joint-specific RA pathology (Table 2), patients treated with bDMARDs had a significantly greater proportion of elbow diagnoses (12.9% versus 10.1%, *P* < 0.001), whereas patients not treated with bDMARDs had a significantly larger proportion of shoulder diagnoses (39.6% versus 43.1%, *P* < 0.001). When evaluating risk of TJA within 2 years after an initial joint-specific RA diagnosis among bdMARD-treated patients versus nbDMARD patients with RA, the patients not treated with bDMARDs exhibited significantly lower risk of THA (13.96% versus 16.32%; OR 0.67; 95% CI 0.46 to 0.98), TKA (10.28% versus 13.38%; OR 0.66; 95% CI 0.54 to 0.82), and all TJA (5.92% versus 7.73%; OR 0.73; 95% CI 0.63 to 0.85) compared with patients treated with bDMARDs (Table 2). Rates of TSA and TEA were statistically comparable between the cohorts (both *P* > 0.05).

**Table 2:** Risk of TJA in Patients Who Were and Were Not Taking bDMARD

Procedure	bDMARD Treatment n (%)^a^	No bDMARD Treatment n (%)^a^	OR	95% CI
All TJA	260 (7.7)	795 (5.9)	**0.73**	**0.63-0.85**
TSA	29 (2.3)	101 (1.8)	0.73	0.46**-**1.19
TEA	12 (2.8)	22 (1.6)	0.52	0.26**-**1.35
THA	63 (16.3)	231 (14.0)	**0.67**	**0.46-0.98**
TKA	162 (13.4)	482 (10.3)	**0.66**	**0.54-0.82**

bDMARD = biologic disease modifying anti-rheumatic drug, CI = confidence interval, OR = odds ratio, TEA = total elbow arthroplasty, THA = total hip arthroplasty, TJA = total joint arthroplasty, TKA = total knee arthroplasty, TSA = total shoulder arthroplasty

a Percentages calculated of the total number of patients in each group (All TJA) or the number of patients from each group with pathology in the given joint.

Reference group, No bDMARD treatment.

## Discussion

Before the implementation and optimization of DMARDs in the RA treatment regimen, there was a high prevalence of joint degeneration, severe long-term disability (19%), and mortality (35%).^5^ Once optimal dosing of nbDMARDs, such as methotrexate (MTX), and development of effective combination therapies were used, patient symptoms and functionality greatly improved.^1^ Current therapies can achieve remission and lower disease activity in up to 75% to 80% of patients.^1,14^ This advancement in treatment has affected orthopaedic surgery profoundly, with the utilization of TJA among patients with RA drastically declining on initiation of this therapy. Jämsen et al^15^ reported a notable decrease in TJA attributed to RA between 1995 and 2010, with TSA decreasing from 66% to 11%, TEA decreasing from 84% to 49%, THA decreasing from 6% to 2%, and TKA decreasing from 12% to 3%. In addition, Louie and Ward^8^ compared the volume of TKA and THA procedures in patients with RA aged 40 to 59 years in 1983 to 1987 with similarly aged patients in 2003 to 2007 demonstrating a 19% and 40% decrease in TKA and THA, respectively. The drastic decrease of TJA in patients with RA was likely directly correlated with the increased use of nbDMARDs, mainly MTX, and the introduction of bDMARDs.

In 2008, the ACR recommended a combination of TFNai and MTX treatment in patients with early RA, high disease activity at <6 months, or TFNai treatment in RA patients with established (>6 months) high disease activity that previously failed MTX.^12^ This recommendation transitioned to an option for TFNai with or without MTX for early RA high disease, with nbDMARD double or triple therapy being an alternative option in 2012 guidelines.^10^ The 2015 ACR guidelines did not recommend bDMARD therapy in moderate-to-high disease until failure of nbDMARD monotherapy with no absolute indication until the failure of combination therapy.^11^ Established RA disease recommendations also transitioned in 2015 to the requirement of MTX failure before giving the clinician the choice of adding another nbDMARD or a TNFai to MTX therapy.^10-12^ Similar to the 2012 and 2015 ACR guidelines, the most recently published ACR guidelines in 2021 strongly recommend MTX monotherapy over bDMARD monotherapy in DMARD-naïve patients with moderate-to-high disease activity.^9^ In contrast to the 2008 ACR guidelines, the 2021 guidelines also conditionally recommended MTX monotherapy over MTX in combination with TNFai for DMARD-naïve patients with high disease activity.^9,12^

In contrast to the reported downtrend in TJA utilization among patients with RA before 2010, our data show a reversal of this trend between 2010 and 2019 with patients with RA experiencing a statistically significant increase in the volume of TEA, TSA, THA, and TKA procedures. The most notable change in TJA volume during this period was the more than two fold increases in utilization observed from 2015 to 2016. This uptrend in TJA utilization among patients with RA aligns temporally with the 2015 change in ACR guidelines, which narrowed indications for initiating bDMARD treatment in patients with RA. Although other factors may have played a role, such as increased overall TJA utilization by the general population or the expansion of Medicaid by some states the same year, this stark increase in TJA volume after 2015 suggests that decreased utilization of bDMARDs was a possible contributor to the increased rates of TJA in this population.^16,17^

This hypothesis is also supported by our secondary analysis on risk of TJA within 2 years after an initial joint-specific RA diagnosis for patients with RA treated versus not treated with bDMARDs. Only 20% of identified patients were treated with bDMARDs, which illustrates adherence to ACR treatment guidelines, given that the period encompassed by the secondary analysis occurred after bDMARD indications were narrowed. Our results showed that patients with RA treated with bDMARDs had an increased risk of THA, TKA, and overall TJA compared with untreated patients, which contradicted our original hypothesis that was based on the demonstrated efficacy of bDMARDs in preventing joint degeneration. However, it is possible that many of the patients treated with bDMARDs did not receive bDMARD treatment until failure of nbDMARDs therapy and possibly combination therapy per 2012 and 2015 ACR guidelines.^10,11^ Therefore, patients with RA treated with bDMARDs likely experienced recalcitrant and/or severe disease for an extended period before initiating bDMARD treatment. This extended period of active disease can lead to destruction of cartilage and bone because of continued inflammation. It is possible that potential therapeutic effects of bDMARD therapy were limited in these patients with significant joint degeneration before starting bDMARD treatment, which may explain the higher risk of TJA relative to patients not treated with bDMARDs.

Despite the known impedance of bDMARDs on joint degeneration, current ACR guidelines do not recommend bDMARD treatment until failure of nbDMARDs because of associated complications (i.e., malignancy and infection) and high cost. Contrary to this belief, a meta-analysis by Ramiro et al^18^ shows that no increased risk of malignancy when comparing bDMARD use in patients with RA with the general population was observed. The same study reported patients with RA treated with TFNais may have slight increased risk of lymphoma when compared with the general population and melanoma when compared with patients with RA treated with nbDMARDs; however, no overall increased risk of mortality was observed with bDMARDs usage in patients with RA. In addition, Singh et al conducted a meta-analysis examining the risk of serious infection among patients with RA treated with bDMARDs compared with patients treated with nbDMARDs and reported an increased risk of serious infections in patients with standard and high-dose bDMARD treatment (OR = 1.52; absolute increase of 6 per 1000). However, the authors also reported no statistically significant difference when bDMARD was used as a monotherapy (MTX-naïve patients) and no increased risk in low-dose bDMARD utilization (OR = 0.93).^19^ Furthermore, Harty et al^20^ demonstrated a significant reduction of hospital costs associated with inpatient stays in patients on TFNαis, a finding which is in part due to decreased TJA utilization. Pooled data imply that judicious utilization of low-dose bDMARD therapy as a first-line treatment for early RA disease may represent a safe option for patients with RA. However, more clinical studies are needed to better understand the potential of bDMARDs in joint preservation.

There are multiple limitations in this study. PearlDiver is a pooled data administrative claims database, limiting access to patient information including previous treatment modalities, radiologic disease severity, and patient functionality. Because the PearlDiver database only provides data on a specific group of patients during a certain period, sampling bias is present. In addition, the use of joint-specific ICD-10 RA codes and requiring continuous database enrollment limited our time interval for the bDMARD treatment analysis to patients with joint-specific RA diagnoses between 2015 and 2018 Q3, and this time limitation may misrepresent the true effect of ACR guideline recommendations. Although the rates of prescription drug claims filed within the database may be accurately quantified, it is impossible to accurately ascertain proper medication utilization and consumption, which may have influenced our results. The effect of differences in access to RA treatment (e.g., financial barriers) is also unclear. With the complex nature of medical billing, there is a possibility of coding bias through manual entry of diagnosis/procedural codes. Although these errors are inherent with any database study using administrative claims data, a recent study by the Center for Medicare and Medicaid Services demonstrated that billing errors made up only 1.0% of overall payments in 2019.^21^ By excluding patients younger than 40 years, some patients who underwent TJA before the age of 40 years were excluded. However, only 1.4% of patients with RA who underwent arthroplasty between 2010 and 2021 Q1 were younger than 40 years in the database. It is possible that some patients could have been prescribed a bDMARD but later switched to a nbDMARD, and these patients would be missed in the analysis. However, in the 2015 guidelines, for example, there is no mention of stepdown therapy from bDMARDs back down to nbDMARDs. Even if the disease activity decreases to ‘low activity,’ the ACR recommended continuing the medication that the patient was already on rather than switching or discontinuing, so this stepdown is likely to represent a small number of patients.^11^ Although not in the scope of the analysis, it is likely many patients with RA underwent TJA at a longer-term follow-up period and would, therefore, not be included in this study. In addition, patients with RA may undergo TJA at younger ages and as such some patients younger than 40 years may have been missed in the analysis. The demand for TJA has increased over the past decade and is predicted to continue increasing.^16,17,22^ As such, this increasing demand may have an effect on TJA utilization in patients with RA. Finally, although we report strong correlations for increased TJA utilization and the revised ACR guidelines, it must be acknowledged that causation cannot be definitively proven given that this study is retrospective and observational.

## Conclusion

Volume of TJA procedures conducted in patients with RA has trended markedly upward over the past decade, with a sharp increase after 2015, which may be partially explained by the decreased utilization of bDMARD treatment in accordance with the most recent ACR guidelines. Patients treated with bDMARDs exhibited an increased risk of THA, TKA, and all TJA within two years after an initial joint-specific RA diagnosis relative to patients with RA without bDMARD treatment. In light of the ACR guidelines, these data likely reflect skewed utilization of bDMARDs in RA patients with more severe disease and prolonged periods of joint degeneration because of prior treatment failure. Future prospective studies are needed to evaluate the efficacy of bDMARDs in slowing or preventing joint degeneration and overall risk of TJA among patients with RA.
